# Exceptional in-plane and interfacial thermal transport in graphene/2D-SiC van der Waals heterostructures

**DOI:** 10.1038/s41598-020-78472-2

**Published:** 2020-12-16

**Authors:** Md. Sherajul Islam, Imon Mia, Shihab Ahammed, Catherine Stampfl, Jeongwon Park

**Affiliations:** 1 Department of Electrical and Electronic Engineering, Khulna University of Engineering and Technology, Khulna, 9203 Bangladesh; 2 School of Physics, The University of Sydney, Sydney, NSW 2006 Australia; 3 Department of Electrical and Biomedical Engineering, University of Nevada, Reno, NV 89557 USA; 4 School of Electrical Engineering and Computer Science, University of Ottawa, Ottawa, ON K1N 6N5 Canada

**Keywords:** Materials science, Nanoscience and technology

## Abstract

Graphene based van der Waals heterostructures (vdWHs) have gained substantial interest recently due to their unique electrical and optical characteristics as well as unprecedented opportunities to explore new physics and revolutionary design of nanodevices. However, the heat conduction performance of these vdWHs holds a crucial role in deciding their functional efficiency. In-plane and out-of-plane thermal conduction phenomena in graphene/2D-SiC vdWHs were studied using reverse non-equilibrium molecular dynamics simulations and the transient pump-probe technique, respectively. At room temperature, we determined an in-plane thermal conductivity of ~ 1452 W/m-K for an infinite length graphene/2D-SiC vdWH, which is superior to any graphene based vdWHs reported yet. The out-of-plane thermal resistance of graphene → 2D-SiC and 2D-SiC → graphene was estimated to be 2.71 × 10^−7^ km^2^/W and 2.65 × 10^−7^ km^2^/W, respectively, implying the absence of the thermal rectification effect in the heterobilayer. The phonon-mediated both in-plane and out-of-plane heat transfer is clarified for this prospective heterobilayer. This study furthermore explored the impact of various interatomic potentials on the thermal conductivity of the heterobilayer. These findings are useful in explaining the heat conduction at the interfaces in graphene/2D-SiC vdWH and may provide a guideline for efficient design and regulation of their thermal characteristics.

## Introduction

The Joule heating generated by electrical current is a key challenge in nanoelectronic and optoelectronic devices, which results in a high power density and spatial heat localization. Such concentrated heat, if not effectively eliminated, could accumulate in thermal hot spots, reduce the overall electronic current density, and lead to significant material failure. Graphene has been suggested as superb material for heat management in nanoelectronic devices because of its exceptional thermal conductivity^1–3^ as well as exotic electronic^4^ and mechanical properties^5^. However, the zero intrinsic bandgap restrains graphene applications to transistor and logic devices. Thus, extensive efforts were made in the last few years to obtain a tunable bandgap along with intrinsic good thermal conductivity. The hybridization of graphene with finite-bandgap semiconductors would solve this limitation and give rise to new potential applications. In this context, van der Waals heterostructures (vdWHs) made from various two dimensional (2D) materials in a single stack attracted great attention due to their unparalleled platforms for exploring new physics, a novel design of devices as well as exotic electrical and optical properties^6–8^. Several graphene-based vdWHs including graphene/h-BN, graphene/MoS_2_, graphene/MoSe_2_, graphene/stanene, graphene/silicene, graphene/phosphorene, graphene/germanene with exciting electronic and optical properties have been explored^9–16^.

Although graphene based vdWHs offer remarkable possibilities in band-gap engineering and improve electronic and optical properties, nevertheless, the choice of a compatible substrate is an important prerequisite to experimentally synthesize these vdWHs. Recent literature revealed that silicon carbide (SiC) is the most appealing substrate for the epitaxial synthesis of graphene^17–19^. On the other hand, a quasi-2D SiC flake has been synthesized by Lin et al.^20^ recently via the thermochemical reaction between Si powder and exfoliated graphene at high temperature. A thin atomic structure with hexagonally bonded SiC nanograins has also been fabricated by Susi et al.^21^. Moreover, 2D-SiC offers a wide direct bandgap with exotic thermal, electrical, optical, and mechanical properties^22–27^. Extraordinary tunable electronic properties have also been demonstrated using 2D-SiC as a promising substrate in stanene/2D-SiC and bismuthene/2D-SiC vdWHs^28,29^. The epitaxial growth of good-quality graphene from SiC and the latest productive efforts to synthesize 2D-SiC greatly inspired the development of graphene/2D-SiC vdWHs as well. Using 2D-SiC as a compatible substrate a wide band-gap of ~ 30 meV along with a well-preserved Dirac cone and tunable electronic properties has been reported for graphene/2D-SiC vdWHs very recently^30^, which could be promising for nanoelectronic and optoelectronic applications. It is intriguing to know whether this heterostructure may also succeed to retain the exotic thermal conductivity of graphene. Understanding the thermal behavior of these materials is thus of fundamental importance in the evolution of high-performance nanoelectronic devices.

Prior investigations demonstrated that the interface thermal conductivity in graphene related carbon systems is very low due to the substrate effects, which is the main constraint for efficient thermal transport and heat dissipation^31^. Thermal properties of the stacked structures can be very dissimilar from its constituents^32,33^; they not only dependent on the choice of 2D materials used for its construction but are also considerably modified by the orientation of the two lattices. Although in-plane lateral thermal conductivity of graphene and 2D-SiC is well known, nevertheless, the mechanism of cross-plane thermal transport in graphene/2D-SiC vdWHs requires deep knowledge before they can effectively compete in real-world technology. The phonon-mediated both intra and interlayer heat transfer needs to be answered to clarify the detailed heat dissipation phenomena of this prospective heterobilayer. However, despite the promising electronic features in graphene/2D-SiC, there is no study on the thermal transport behavior of this heterobilayer.

In this work, we investigated thoroughly the in-plane and cross-plane heat conduction in graphene/2D-SiC vdWHs by means of molecular dynamics (MD) simulations. The lateral thermal conductivity was calculated via the reverse non-equilibrium molecular dynamics (RNEMD) technique and the cross-plane thermal conductivity was estimated utilizing the transient pump-probe (TPP) technique. To clarify the heat dissipation phenomena between graphene and 2D-SiC layers, phonon properties of the graphene/2D-SiC heterobilayer as well as individual graphene and 2D-SiC were estimated utilizing the velocity correlation of atoms. The calculated in-plane thermal conductivity for the graphene/2D-SiC heterobilayer is outperformed comparable to any graphene based heterostructures reported yet. The thermal resistance at the interface in both the graphene to 2D-SiC and 2D-SiC to graphene directions demonstrated that the heterobilayer has no thermal rectification effect. This study provides a profound intuition into the heat management for nanoelectronics based on the graphene/2D-SiC heterobilayer.

## Methods

We employed 12–6 Lenard–Jones (LJ) potential to model the vdW interaction between graphene and the 2D-SiC layer which can be represented as:

1 $$V\left( r \right) = 4\chi \varepsilon \left[ {\left( {\frac{\sigma }{r}} \right)^{12} - \left( {\frac{\sigma }{r}} \right)^{6} } \right];\quad {\varvec{r}} < {\varvec{r}}_{{\varvec{c}}}$$

Here *ε*, *r* and *σ* denote the energy, the distance between two atoms, and the atomic length parameters, respectively. *χ* signifies the interface interaction parameter that can be adjusted to modulate the coupling strength. These parameters were considered as ${\varepsilon }_{C-C}\hspace{0.17em}$= 4.560 meV, ${\varepsilon }_{Si-C}$ = 8.909 meV, ${\sigma }_{C-C}$ = 3.431 Å and ${\sigma }_{Si-C}\hspace{0.17em}$= 4.067 Å, based on the universal force field established by Rappe et al.^34^. The interface interaction parameter was fixed as *χ* = 1 for default interaction strength. For all vdW interactions, we set the cutoff distance of the LJ potential as 10 Å. The initial spacing between graphene and the 2D-SiC sheet was set at 3.4 Å, which can be further modified automatically under the relaxation of the structure. To remove the edge effects, periodic boundary conditions were used in both in-plane (*x* and *y*) and out-of-plane (*z*) directions. The atomic interaction with the system image was eliminated by setting a high vacuum distance as 10 nm in the out of-plane direction. We set a time step of 0.5 fs in all MD simulations. All the calculations were conducted by means of Large Scale Atomic/Molecular Massively Parallel Simulator (LAMMPS) package^35^. We employed the optimized Tersoff potentials implemented by Lindsay et al.^36^ and Albe et al.^37^ to interpret the interactions between C–C within the graphene structure and Si–C within the 2D-SiC sheet, respectively. These potentials were successfully implemented to predict the thermal behavior of graphene and 2D-SiC precisely.

**Figure 1:**
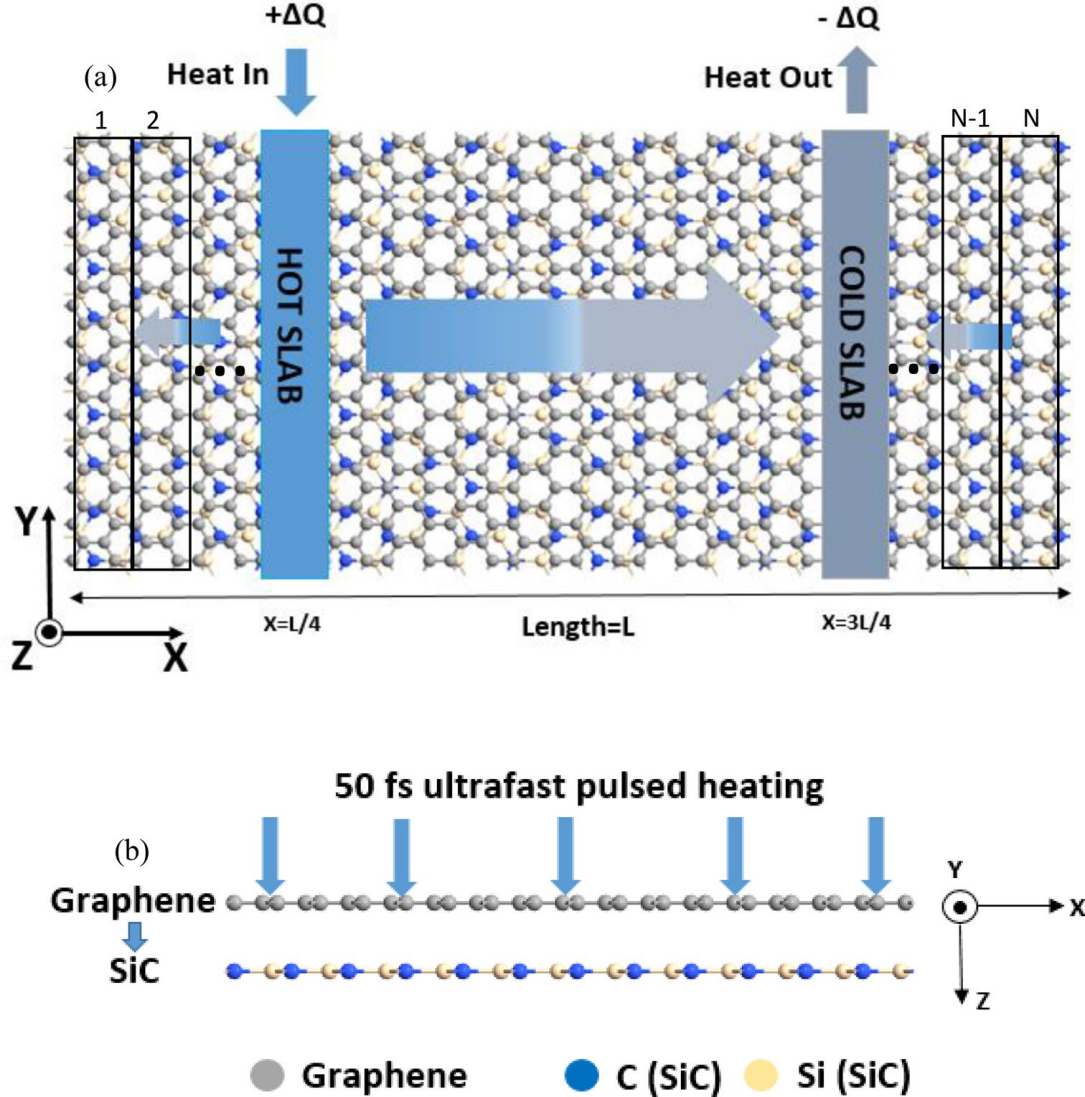
Atomic configuration of the graphene/2D-SiC van der Waals heterostructure (vdWH) used to calculate the (**a**) in-plane and (**b**) out-of-plane thermal conductivity. The + *∆Q* amount of heat is applied to the hot slab (blue area) placed at the length of *X* = *L/4* and *− ∆Q* amount of heat is extracted from the cold slab (gray area) placed at the length of *X* = *3L/4* in the x direction. Here, the blue to gray gradient arrow reflects the heat flow around the sheet length. A 50 fs ultra-fast heat impulse is applied to the graphene in the out-of-plane (*z*) direction to compute the interface thermal resistance.

Throughout the analysis, the RNEMD simulations^38^ were employed to compute the in-plane thermal conductivity of the graphene/2D-SiC heterobilayers. The RNEMD technique is a straightforward approach where the thermal conductivity is determined by applying a heat flux as the input and calculating the temperature gradient as the output according to the Fourier's law:

2 $${k}_{x}=-\frac{{j}_{x}}{dT/dx}$$

where ${j}_{x}$_,_ *dT/dx,* and ${k}_{x}$ represent the heat flux, the temperature gradient and the thermal conductivity along the *x* direction, respectively. A hot and cold slab both of widths 20 Å were placed in the heterobilayer structure at the *L*/*4* and *3L*/*4* positions, respectively, where *L* denotes the heterobilayer length in the *x* direction. To establish the temperature difference between hot and cold regions, the velocity rescaling method for thermal energy modulation of the atoms was implemented. Thus, the heat flux flowing through the material can be expressed as ${J}_{x}=\frac{\Delta\upvarepsilon }{2AMt}$, where, *Δε* denotes the disparity in thermal energy between the hot and cold atoms in every time step *M (M* is classified as exchange frequency of heat energy). *t* is the MD time step, *A* = *wh*, the cross-sectional area of the system. The factor 1⁄2 was incorporated in the equation because of the system’s periodicity which means that heat energy will flow in both directions. The temperature gradient was calculated by dividing the whole sheet into *N* slabs along the computational direction (*x*) as illustrated in Fig. 1, where each slab having width 10 Å. The temperature at any slab ‘*s*’ can be calculated by using,

3 $${T}_{s}= \frac{\sum_{a=1}^{{N}_{s}}{m}_{a}{{v}_{a}}^{2}}{3{N}_{s}{k}_{B}}$$

where ${N}_{s},{K}_{B},{m}_{a}$ and ${v}_{a}$ represent the total number of atoms at slab ‘*s*’, the Boltzmann constant, mass and velocity of atom ‘a’ in the slab, respectively.

We conducted our simulation in two steps: structure relaxation and heat-imposing steps. At relaxation step, the system was first set to an equilibrium state at the target temperature. Following energy minimization of the structures via Conjugated Gradient Algorithm, isothermal-isobaric (NPT) ensemble was conducted for 10^5^ time steps to adapt the heterobilayer to the preferred temperature and pressure. To achieve the target temperature, the velocity-verlet integration approach was employed. We then applied NVE MD simulations for 10^5^ time steps to raise the initial temperature to the desired temperature. To stabilize the temperature and pressure as well as to preserve the volume and energy of the heterobilayer, 2 × 10^5^ time steps of NPT and 3 × 10^5^ time steps of NVE MD simulations, respectively, were conducted. Before applying the heat energy, we again placed $5\times {10}^{5}$ time steps of NVE ensemble for more stabilizing the temperature, conservation of energy, and maintaining the steady-state condition. The distributions of atomic velocity in graphene and 2D-SiC were analyzed quantitatively and compared with the predictions of Maxwell–Boltzmann distributions to justify the system being a stable state. If the system becomes stable, the atomic velocities within the heterobilayer should meet the distribution of Maxwell–Boltzmann prediction as:

4 $${P}_{M }=4\pi {v}^{2}{\left(\frac{m}{2\pi {K}_{B}T}\right)}^\frac{3}{2}{e}^{-\frac{m{v}^{2}}{2{K}_{B}T}}$$

Here *m*, ${K}_{B}$ and ${P}_{M}$ represent the mass, the Boltzmann constant, and the probability of an atom with velocity *ν*. Besides, to check the stability of the system temperature, we also record the system temperature as a function of time. If the system temperature remains constant over time, reflecting the equilibrated state of the system at that temperature. Finally, after imposing the heat energy, the system was run another $24\times {10}^{6}$ time steps of NVE to achieve the time-averaged temperature distribution from which $dT/dx$ is measured.

To obtain the out-of-plane thermal transport, we calculated the interfacial thermal resistance using the TPP technique^32^. This method was successfully used to study the out-of-plane thermal transport of different 2D heterostructures^33,39,40^. After equilibrating the system at designated temperatures, a 50 fs ultrafast thermal impulse was applied to the graphene layer. The graphene temperature rises rapidly during the ultrafast thermal impulse of 50 fs, while the 2D-SiC temperature is set at 300 K according to the TPP technique. After 50 fs, the graphene temperature started rapidly decreasing, and the 2D-SiC temperature rises gradually as the ambient environment is a vacuum. The heat energy will dissipate by means of interlayer heat exchange before a new thermal equilibrium is achieved by the graphene/2D-SiC bilayer. The total energy of graphene and the changes of temperature in each layer were noted at each time step. The variation of energy will obey the equation below:

5 $$\frac{\Delta {E}_{t}}{\Delta t}=\frac{{A}_{R} \times ({T}_{graphne}-{T}_{2D-SiC })}{R}$$

where *E*_*t,*_* t*, and *A*_*R*_ denote the total energy, time and contact area, respectively. The temperature in the heat applied layer and the unimpeded layer is indicated by *T*_*graphene*_ and *T*_*2D-SiC*_, respectively. With the least squares approach we used an integral formula to calculate the optimal *R* value as^39,40^:

6 $${E}_{t}={E}_{0}+\left(\frac{{A}_{R}}{R}\right).{\int }_{0}^{t}\left({T}_{graphne}-{T}_{2D-SiC }\right)dt$$

where ${E}_{o}$ is the initial energy. After calculating the integral ${\int }_{0}^{t}\left({T}_{graphne}-{T}_{2D-SiC }\right)dt$, a linear relation can be obtained between *E*_*t*_ and this integral, in which the slope is ${A}_{R}$*/R*. The out-of-plane thermal resistance *R* at the interface can be found on the basis of the contact area *A*_*R*_. We also analyzed the thermal rectification effect on *R*. The heat transport from graphene to 2D-SiC is denoted as graphene → 2D-SiC, and the heat transport from 2D-SiC to graphene is denoted as 2D-SiC → graphene, respectively. To obtain noise-free data points, the collected data points are averaged at every 100-time steps. To eliminate the statistical errors, we conducted three separate simulations with specific initial conditions for both in-plane and cross-plane thermal conductivity calculations. Through combining each simulation, the final values were determined using the standard deviations displayed as an error bar.

## Results and discussion

**Figure 2:**
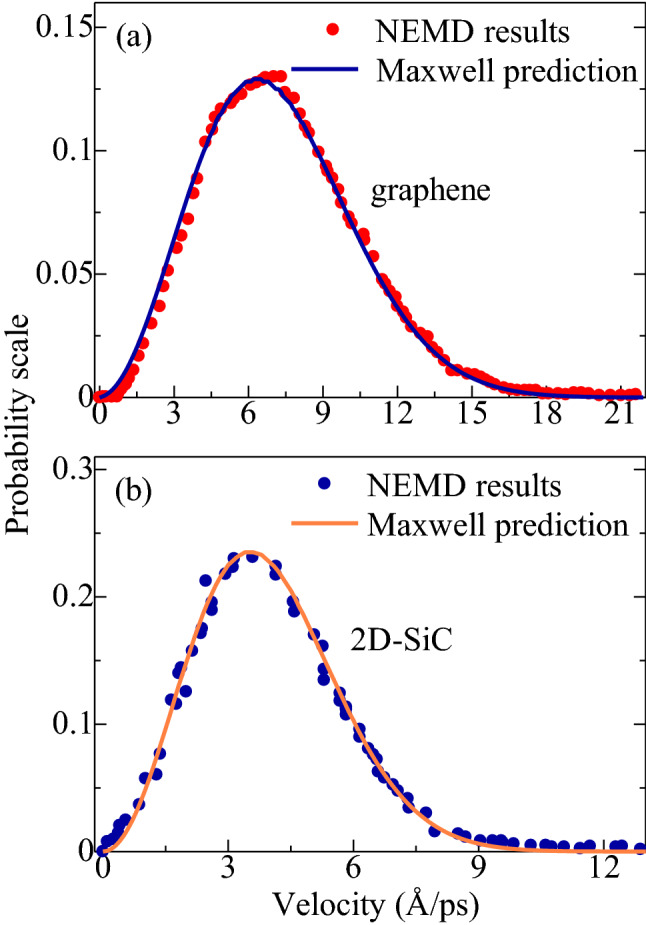
Comparison of the atomic velocity distribution of (**a**) graphene and (**b**) 2D-SiC layers with Maxwell prediction at steady-state.

SiC is considered as one of the most important substrates to synthesize and use with graphene for technological applications. Moreover, 2D-SiC has been shown to enable graphene to retain the exotic electronic features, as well as to introduce a direct bandgap creation in the heterobilayer^30^. It is therefore of great general concern to probe the thermal behavior of this heterostructure and to know whether the unique thermal conductivity of graphene is still preserved. The RNEMD simulation was used to probe the in-plane thermal behavior of this appealing heterobilayer. Before applying the RNEMD simulation, the steady-state condition of the system was confirmed by estimating the atomic velocities of the graphene and 2D-SiC layers at thermal equilibrium. Figure 2 illustrates the comparison of the velocity distribution obtained from the RNEMD simulation and the Maxwell theoretical estimations as defined in Eq. (4). It is observed that the predicted distributions are perfectly matched to the MD simulation outputs, suggesting that the heterobilayer attains a stable state.

**Figure 3:**
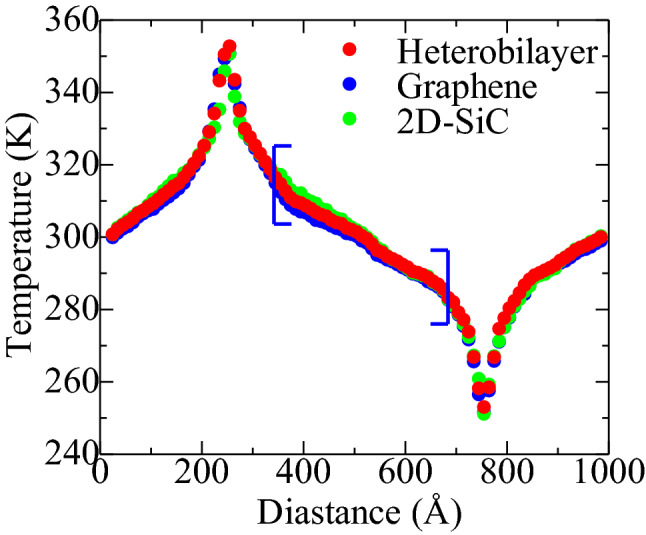
Temperature distribution profile of graphene, 2D-SiC, and the graphene/2D-SiC heterobilayer at steady-state and 300 K. Here the length and width of the graphene/2D-SiC heterobilayer are 100 nm and 10.8 nm, respectively. In hot and cold areas, temperature differences are approximately 300 ± 50 K.

To characterize the thermal conductivity, the temperatures of hot slab and cold slab are monitored as *T* + *∆T* and *T − ∆T*, respectively, where a *∆T* of 50 K is considered. The temperature profile of the heterobilayer having the area of 100 × 10.8 nm^2^ (length × width) at the steady-state condition is shown in Fig. 3. A linear drop of temperature can be perceived from the hot area to the cold area. Earlier RNEMD simulations also reported^23,24,42–44^ similar phenomena for various heterobilayers as well as 2D material systems. The thermal conductivity was calculated by taking the temperature gradient, which was obtained from linear fitting. Temperature profiles adjacent to the hot and cool regimes were exempt from the linear fitting process due to the incessant interchange of energy in the heat sinks, where phonon scattering causes an increase in nonlinearity. The temperature gradient *∇T* was determined using only the linear region as shown by the blue square bracket in Fig. 3. The temperature profile of individual graphene and 2D-SiC sheets are displayed in Fig. 3 for comparison. It is perceived that the temperature profile of the heterobilayer remains within the graphene and 2D-SiC profiles. The estimated *∇T* for graphene, 2D-SiC and the heterobilayer are 0.081762 K/nm, 0.096669 K/nm and 0.087131 K/nm, respectively.

**Figure 4:**
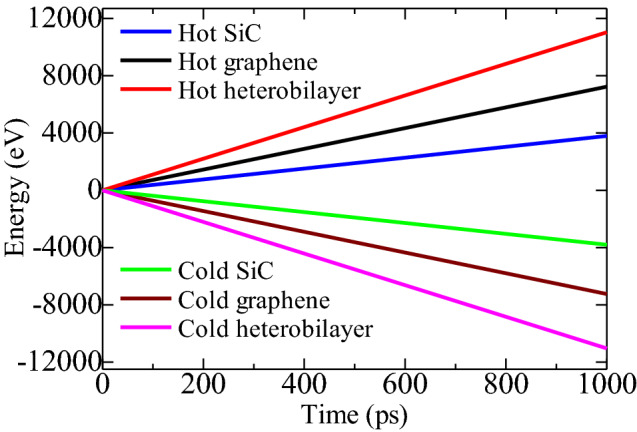
Aggregated energy added to the hot region and extracted from the cold region as a function of time.

In addition to the temperature gradient, the amount of heat flux is also required to calculate the thermal conductivity utilizing Fourier’s law. The evolution of thermal energies in the hot and cold regimes of the heterobilayer is portrayed in Fig. 4. The thermal energies show a linear relationship with simulation time. The energy required to preserve a 100 K temperature gradient in graphene is far greater than that of 2D-SiC, suggesting that graphene has better thermal transport capacity. This also indicates that the thermal energies of graphene will be dissipated much faster from the hot regime to the cold regime. The heat flux in each system can be calculated from the slope of the energy profile. The computed linear fitting slopes for graphene and 2D-SiC are 15 eV/ps and 3.17 eV/ps, respectively. Consequently, the estimated thermal conductivities for $$100\times 10.8$$ nm^2^ graphene ($${k}_{G})$$, 2D-SiC ($${k}_{2D-SiC})$$ and the heterostructure ($${k}_{G/2d-SiC})$$ are 812.2113 W/m-K, 145.177 W/m-K, and 451.616 W/m-K, respectively. The obtained thermal conductivities of graphene and 2D-SiC are in line with former literature^1,23,44^. Moreover, the calculated thermal conductivity of the graphene/2D-SiC heterobilayer shows a quite high value compared to the thermal conductivities of graphene/MoS_2_^45^, graphene/MoSe_2_^46^, graphene/stanene^41^, silicene/graphene^16^ and individual 2D-SiC^23^ of the same length. However, our obtained thermal conductivity is comparable with the thermal conductivity of graphene/C_3_N^43^.

Usually, the size of the system has a substantial impact on the calculated thermal conductivity in MD simulations. The heterobilayer structure with lengths 22.16, 42.63, 85.25, 170.1, 255.15, 336.80, 428.65, and 555.28 nm were modeled to study the size effects. The widths of all the structures were the same, with the value of 10.8 nm. Moreover, the thickness of the system largely affects the in-plane thermal conductivity. The height of a monolayer is generally considered as the thickness of that particular 2D material^45,47,48^. However, the thickness of a heterobilayer must be carefully chosen since there is a vdW gap between the two monolayers. Throughout the analysis, the total thickness of the heterobilayer was considered to be the summation of two individual monolayer thicknesses. We assumed the thickness of 2D-SiC, *d*_*2D-SiC*_, and graphene, *d*_*G*_ were 3.5 Å and 3.35 Å, respectively^23,49^. Hence, the thickness of the graphene/2D-SiC heterobilayer, *d*_*G/2D-SiC*,_ was 6.85 Å. Nevertheless, Wu et al.^50^ recently suggested that the layer thickness of various 2D structures does not change with variation of the material. They revealed that the graphene thickness (3.35 Å) could be used for measuring thermal conductivity in all types of 2D materials. Accordingly, both *d*_*G*_ and *d*_*2D-SiC*_ are taken to be 3.35 Å. In this context, the overall thickness of the heterobilayer (*d*_*G/2D-SiC*_) is 6.7 Å. We considered both of these thicknesses in this study, which we denoted as vdW thickness (6.85 Å) and unified thickness (6.7 Å).

**Figure 5:**
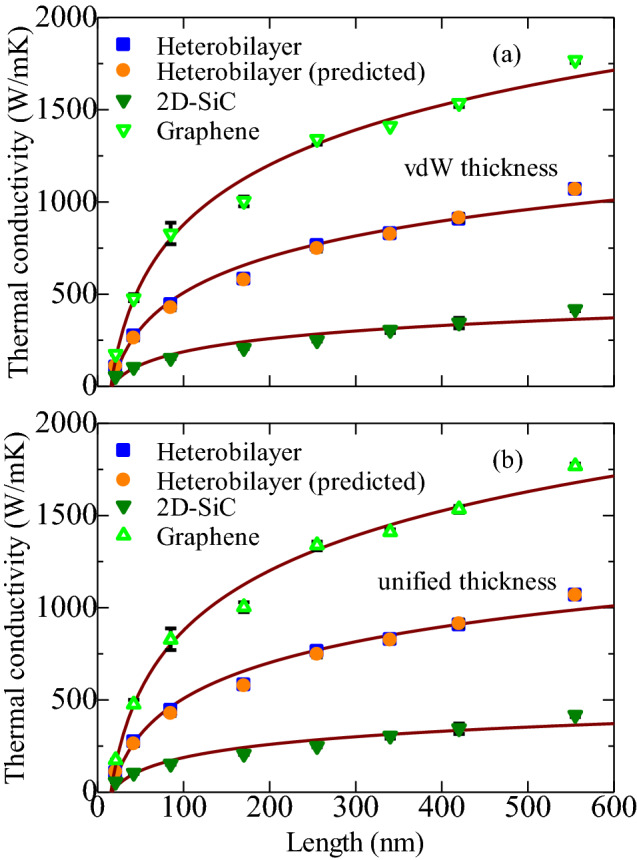
Length dependent in-plane thermal conductivity of graphene, 2D-SiC, and the graphene/2D-SiC heterobilayer with (**a**) vdW thickness and (**b**) unified thickness where the length varied from 22 to 555 nm with fixed-width 10.8 nm. The solid line indicates the theoretically calculated in-plane thermal conductivity of the heterobilayer from Eq. (7) which corresponds well with the RNEMD calculation. Three independent simulations with different initial conditions are used to suppress the statistical errors.

Figure 5 presents the length dependence of the thermal conductivity for graphene, 2D-SiC, and the heterobilayer considering the vdW as well as the unified thickness. The thermal conductivities for both types of thicknesses are almost the same. The in-plane size of experimentally synthesized graphene/2D-SiC heterobilayers should have the ranges from a few nanometers to several micrometers. However, to perform MD simulations on the micrometer scale is still challenging. Thus, based on the simulation findings at the nanoscale, an approximation method is established to estimate the thermal conductivity of graphene/2D-SiC heterobilayers at the microscale. To attain the in-plane thermal conductivity from RNEMD simulations using the Fourier’s law, the heat flux of the system should be correlated. The total heat flux in the heterobilayer is the summation of those from graphene and 2D-SiC. Thus, based on Fourier’s law as described in Eq. (2), correlations of thermal conductivities $k_{G}$, $k_{2D - SiC}$ and $k_{G/2D - SiC}$ can be described using the equation derived by Liu et al.^45^ as:

7 $${k}_{G/2D-SiC}{d}_{G/2D-SiC} \nabla {T}_{BG/2D-SiC}={k}_{G}{d}_{G}\nabla {T}_{G}+{k}_{2D-SiC}{d}_{2D-SiC}\nabla {T}_{2D-SiC}$$

It can be observed from the temperature profiles (Fig. 3) of the graphene/2D-SiC heterobilayer, graphene, and 2D-SiC layers that they overlap each other and their temperature gradients $\nabla \mathrm{T}$ have only 5.32% difference. Thus, we can assume that $\nabla {T}_{BG/2D-SiC}$, $\nabla {T}_{G}$ and $\nabla {T}_{2D-SiC}$ are equivalent, which from Eq. (7) give,

8 $${k}_{G/2D-SiC}{d}_{G/2D-SiC}={k}_{G}{d}_{G}\left(1+\frac{{k}_{2D-SiC}{d}_{2D-SiC}}{{k}_{G}{d}_{G}}\right)$$

Using this empirical relation it is possible to quantitatively obtain the thermal conductivity of a heterostructure accurately and rapidly. The predicted thermal conductivities of the graphene/2D-SiC heterobilayer using Eq. (8) are also shown in Fig. 5 by the orange circles, which coincides well with the RNEMD calculations.

Moreover, with the increased length of the heterobilayer, the thermal conductivity rises monotonously and eventually converges. The thermal conductivities of the heterobilayer, graphene and 2D-SiC rise monotonously from 95.46 to 1065.71 W/m-K, 178.12 to 1780.80 W/m-K and 55.76 to 408.9 W/m-K, respectively, with an increased length of 22.16 to 555.28 nm. It is perceived that the thermal conductivity of heterobilayer lies between graphene and 2D-SiC. An earlier study on graphene and C_3_N by Gao et al.^51^ stated that the overall thermal conductivities are the cumulative result of various phonons with different mean free paths (MFPs). The MFP of phonons change by the scattering of various phonons, which largely relies on the system size as well. When the length of the system increases, phonons with longer MFP take part in the heat conduction, which causes the increase of the in-plane thermal conductivity. If the length of the heterobilayer is lower than the phonon MFP, the phonons will be transported ballistically and the thermal conductivity will rise almost linearly with length. If the length of the system is higher than the MFP of phonon, the phonons will be transported through the system diffusively, which slows down the increasing rate of the thermal conductivity.

The infinite-length thermal conductivity was also calculated via the kinetic theory and Matthiessen's rule by adapting the MD outputs with diverse system sizes^52^. If all phonon modes are considered to have the same average group velocity and MFP similar to a single phonon mode, the thermal conductivity for infinite length can be predicted as:

9 $$\frac{1}{k} = \frac{1}{{k_{\infty } }} \left( {1 + \frac{\Lambda }{L}} \right).$$

**Figure 6:**
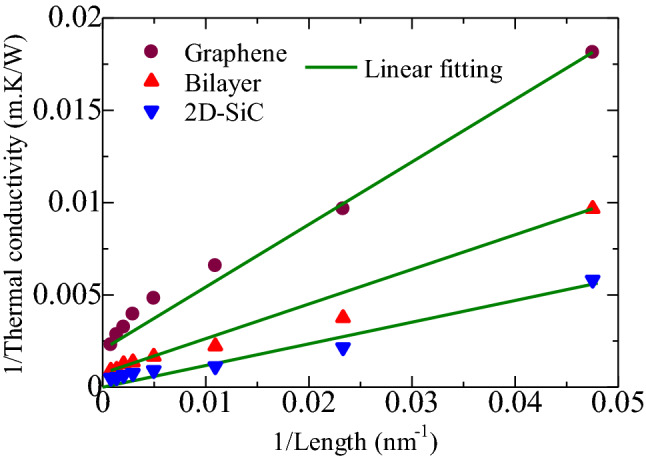
Inverse relation between thermal conductivity and length for extracting infinite length thermal conductivity of graphene, 2D-SiC, and the graphene/2D-SiC heterobilayer.

Here, $k_{\infty } ,\Lambda$, and *L* signify the infinite-length thermal conductivity, the effective MFP of the phonon, and the system length, respectively. Figure 6 depicts the relationship between *1/k* and *1/L*. By performing a linear fitting on the RNEMD results, the infinite-length thermal conductivities of graphene, 2D-SiC and the heterobilayer are 2239.49 W/m-K, 430.19 W/m-K, and 1451.59 W/m-K, respectively. The calculated thermal conductivity for the graphene/2D-SiC heterobilayer outperforms compared to any graphene based heterostructures predicted in the literature, such as graphene/phosphorene, graphene/*h*-BN, graphene/MoS_2_, graphene/MoSe_2_, and graphene/silicene^16,32,33,45,46^. Moreover, to elucidate the interlayer interactions, we have estimated the ${k}_{\infty }$ of the free-standing graphene as well as the 2D-SiC. The predicted ${k}_{\infty }$ values for free-standing graphene and 2D-SiC are 3601.032 W/m-K and 526.789 W/m-K, respectively. Although these values are well matched with the earlier literatures^53,54^, however, they are much higher than the individual supported graphene (2239.49 W/m-K) and 2D-SiC (430.19 W/m-K) in the heterobilayer structure. This suggests that the in-plane thermal conduction decreases in both graphene and 2D-SiC layers due to interlayer interactions. The predicted lower thermal conductivity in graphene is due to the lessening of the flexural (ZA) phonons in graphene/2D-SiC vdWHs. As described by Seol et al.^55^, the main heat carrier in the free-standing graphene is the ZA phonon, which can be scattered and leaked when attached with a substrate. The enhanced phonon scattering between the interlayers reduces the ZA phonons. In free-standing single-layer graphene, in-plane (LA and TA) phonons and out-of-plane phonons are well decoupled. Consequently, there is no chance of enhancing phonon scattering in the out-of-plane direction. In contrast, when graphene stacked with a certain material, then in-plane and out-of-plane phonons are well coupled with supported layer phonons, and different parameters of graphene such as translation and rotation, as well as the supporting layer are also altered. As a consequence, phonon modes in the graphene/2D-SiC heterostructure will also be modified, and phonon scattering through the interlayer will be increased, which will minimize the presence of flexural phonons as well as lateral (LA and TA) phonons. A similar anomaly was also perceived in graphene/C_3_N^43^, graphene/stanene^41^ and graphene/MoS_2_^45^ structures.

**Figure 7:**
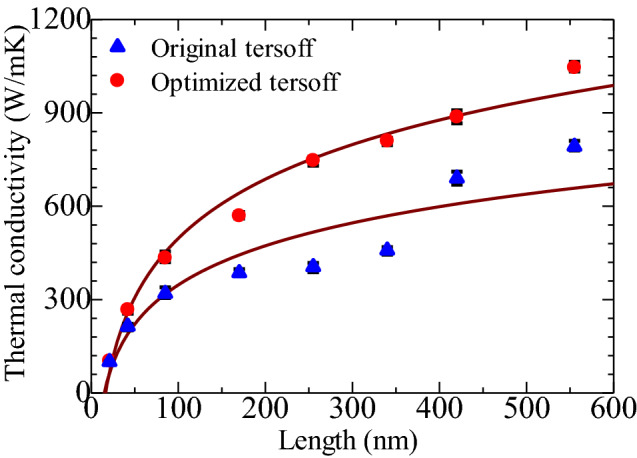
Comparison of the graphene/2D-SiC heterobilayer in-plane thermal conductivity between the original (triangle) and the optimized (circle) Tersoff potential.

In order to determine the thermal conductivity by MD simulations, interatomic potentials are the essential parameter that adequately describes the atomic interactions. Both intralayer and interlayer interactions play a pivotal role in obtaining the thermal conductivity of multilayer graphene and related heterobilayers. Previous studies^56–60^ used several distinct potential models to evaluate the thermal conductivity of monolayer graphene and its ribbon. Amid these potential models, the Tersoff^61–63^ model is a conventional potential built for demonstrating the energetics of covalent structures with classical inter-atomic potentials. Along with the Tersoff potential, the reactive empirical bond order (REBO) potential was also applied to estimate the thermal conductivity of single-^56,64^ and multi-layer^65,66^ graphene. Even though the Tersoff, and extended REBO such as AIREBO potential are often used in carbon-based systems, recently the reactive force field (ReaxFF) potential^67,68^ has drawn great attraction for better performance in doped-graphene, functional graphene oxide, and graphite/polymer nanocomposite interfacial thermal resistance^69,70^. Nonetheless, the improved model of the Tersoff potential, referred to as the opt-Tersoff suggested by Albe et al.^37^ was established mainly for Si, C, and SiC structures and demonstrated that the thermal and phonon characteristics of such materials can be well replicated due to their precise calculation of the energy and interatomic forces. We used both the modified and initial Tersoff potentials to study the impact of parameterization on the thermal conductivity of graphene/2D-SiC heterobilayers. A comparison of the thermal conductivity estimated by the original and optimized Tersoff potentials is shown in Fig. 7. The optimized Tersoff potential shows a high thermal conductivity compared to the original Tersoff potential. The phonon dispersion energy and phonon group velocity calculated by these two potentials are different, which may be attributed to this variation. The original Tersoff potential is stated to underestimate the out-of-plane acoustic phonon, and incorrectly estimate the phonon group velocities and the phonon–phonon scattering near the Brillouin zone (BZ) center. In contrast, the optimized Tersoff potential^37^ precisely generates the phonon dispersion using the analytical model that is confirmed by the first-principles calculations. Thus, the obtained thermal conductivity from the optimized Tersoff potential is more precise due to the enhanced acoustic phonon branches and decreased phonon–phonon scattering intensity along with the best fitting of phonon group velocities at BZ center.

**Figure 8:**
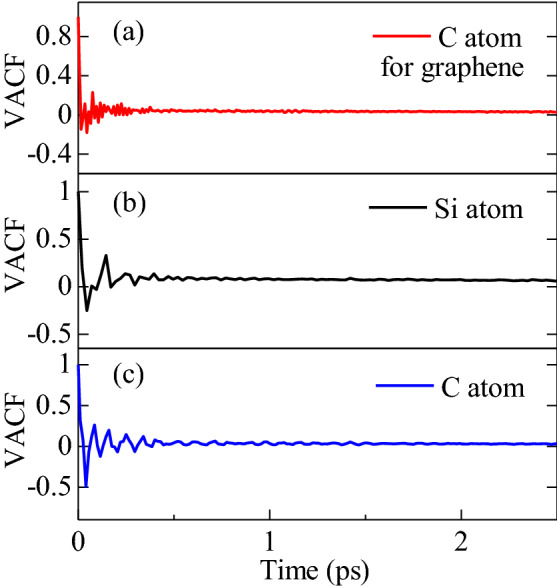
Calculated velocity auto correlation function (VACF) for (**a**) graphene, (**b**) Si, and (**c**) C demonstrating an eroding behavior for a correlation period of 1 ps.

Phonon plays a major role in the heat conduction in 2D materials rather than the electron conduction^71^. Quite different phonon dispersion, phonon density of states (PDOS), and scattering effects are observed in 2D materials compared to their bulk structure, leading to different thermal conductivity with different configurations^72^. With an intension to quantitatively explain the thermal transport process, the PDOS of the heterobilayer is explored. We calculated the PDOS using the Fourier transform of the velocity autocorrelation of atoms^73^. The velocity autocorrelation function (VACF) is formulated as:

10 $${Z}_{\alpha }\left(t\right) = \frac{<{V}_{i\alpha }\left(0\right)\cdot {V}_{i\alpha }\left(t\right)>}{<{V}_{i\alpha }\left(0\right)\cdot {V}_{i\alpha }\left(0\right)>}$$

where the velocity of the *i*th atom of materials *α* at time *t* and time 0 is *v*_*iα*_*(t)* and *v*_*iα*_*(0)*, respectively. The angle brackets represent the ensemble averaging. The PDOS was estimated using the following expression^74^:

11 $$F\left(\omega \right)=\sum_{\alpha }{F}_{\alpha } (\omega )$$

where *F*(*ω*)*, ω,* and ${F}_{\alpha }(\omega )$ are the phonon spectrum, frequency and partial PDOS, in which ${F}_{\alpha }\left(\omega \right)$ is related to the autocorrelation function as *π∫ Zα(t)cos(ωt)dt*. The calculated VACF for the graphene, Si and C atoms are illustrated in Fig. 8a–c, respectively. The VACF provides a greater degree of oscillating characteristic at a low correlation time. Nevertheless, the oscillatory nature diminishes with increasing correlation time, resulting from the interaction between the atoms and the forces from adjacent atoms. Hence, the computed VACF can be employed to explore the phonon states of the system.

**Figure 9:**
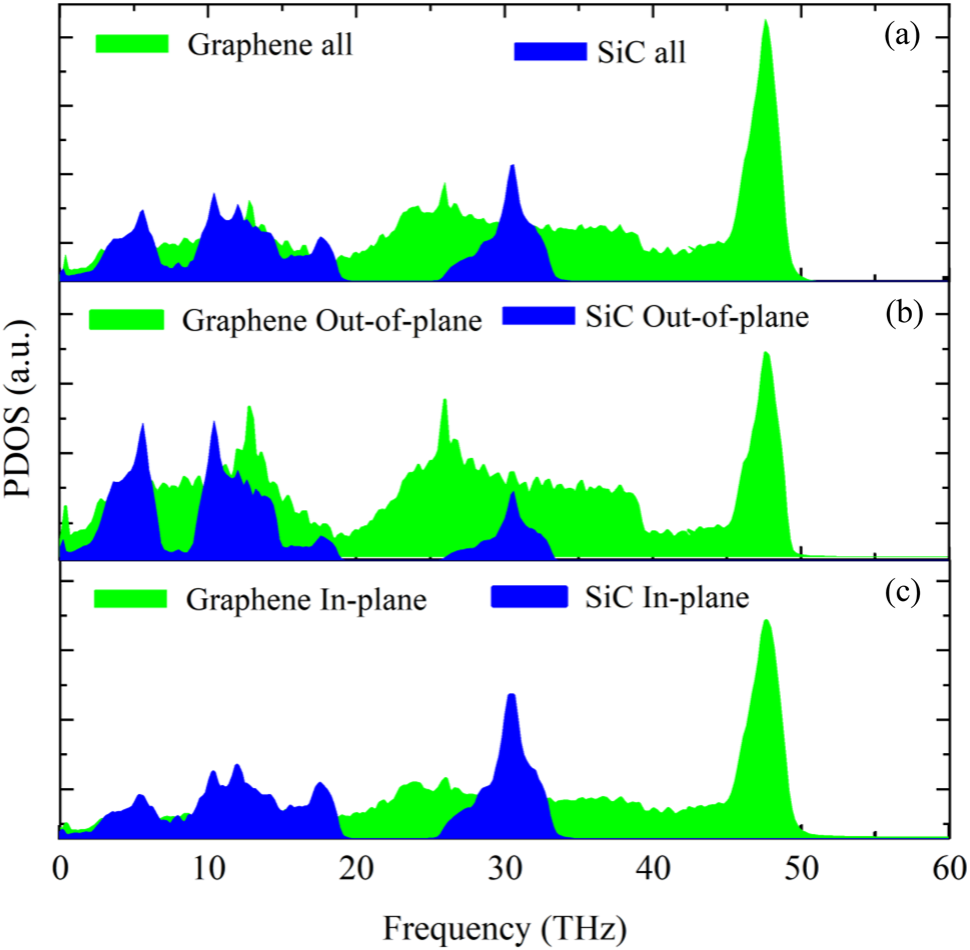
Phonon density of states (PDOS) of (**a**) all (including both in-plane and out-of-plane directions, (**b**) out-of-plane (*Z*) direction, and (**c**) In-plane (*X–Y*) direction for graphene and 2D-SiC.

To show the variations in phonon frequency in graphene and the 2D-SiC layer, the total and decomposed PDOS for in-plane and out-of-plane directions of freestanding samples were calculated. Figure 9a–c displays the total, in-plane longitudinal and transverse, as well as the out-of-plane PDOS for the separate graphene and 2D-SiC monolayer, respectively. The majority of the in-plane phonons in graphene are in the high-frequency regions, whereas out-of-plane phonons are in the low-frequency regimes. The PDOS in 2D-SiC softened towards the low-frequency regime relative to the graphene PDOS. Although it is well established that the thermal transport in graphene is dominated by the in-plane acoustic phonons^75^, recent investigations have presented otherwise. Seol et al.^55^ reported by evaluating the heat transport of supported graphene on amorphous SiO_2_ that the flexural acoustic phonon (ZA) may contribute to the thermal conductivity of suspended graphene as 77% and 86% at 300 K and 100 K, respectively, because of the large mean scattering time of ZA phonons as well as high specific heat. Lindsay et al.^76^ also demonstrated that the ZA phonon makes a dominant contribution to graphene thermal conductivity. Besides, as Fig. 9 implies, the 2D-SiC PDOS is restricted to ~ 35 THz, while the graphene PDOS is limited to ~ 50 THz. This lower PDOS results in lower 2D-SiC thermal conductivity relative to the graphene.

**Figure 10:**
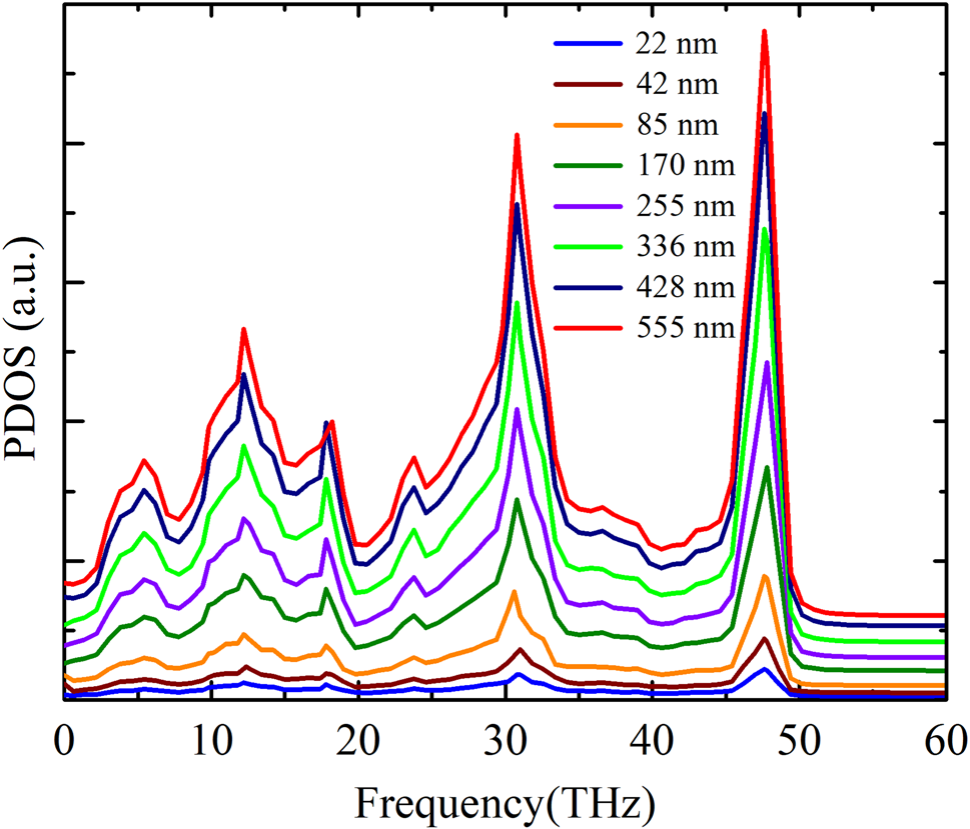
In-plane PDOS of the graphene/2D-SiC heterobilayer for varying lengths from 22 to 555 nm with a fixed-width of 10.8 nm at 300 K.

Figure 10 depicts the length-dependent PDOS of the graphene/2D-SiC heterobilayer. As the sheet length increases, an enhanced behavior is observed at the PDOS peaks. A prior study on freestanding graphene revealed that^77^ the flexural acoustic or ZA phonon contributes insignificantly due to its low group velocity and wide Grüneisen parameter^78^. On the other hand, Lindsay et al.^76^ noticed that most of the heat in graphene is carried by the ZA phonons. At low temperature, the ZA phonon causes a ~ *T*^1.5^ behavior, whereas the LA and TA phonons induce a ~ *T*^2^ behavior of the thermal conductivity. The ZA phonon contributes greatly to the thermal conductivity of compound 2D materials, including 2D-GaN and 2D-ZnO. Moreover, all of these low-frequency phonon states engage in the heat conduction of 2D-SiC^23,24^. Thus, the thermal transport in the graphene/2D-SiC heterobilayer is predicted to result from the contribution of all low-frequency phonon modes. The participation of theses modes also increases with increasing sheet length. As a consequence, the thermal conductivity of the graphene/2D-SiC heterobilayer may be increased with increasing sheet length.

**Figure 11:**
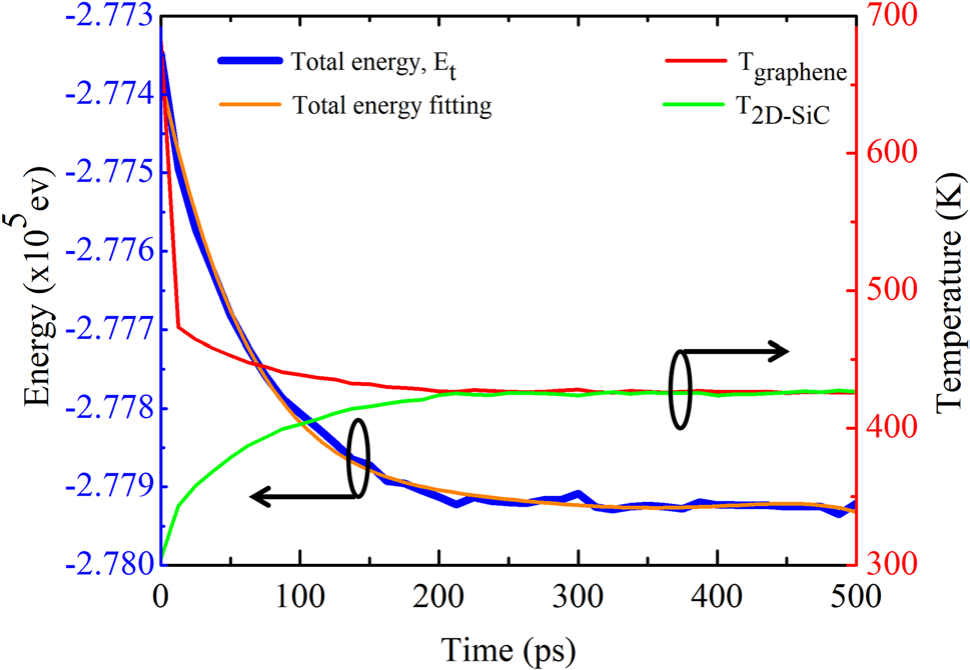
Temperature profiles of graphene and 2D-SiC layers as well as total energy of the graphene layer as a function of time.

Although 2D materials exhibit an outstanding in-plane thermal conductivity, the weak vdW interaction across the interface constrains the out-of-plane heat conduction. To elucidate the out-of-plane thermal energy exchange, we evaluated the thermal resistance at the interface of the heterobilayer. Periodic boundary conditions in the *x*, *y*, and *z* directions are used here to eradicate the size effect. During the thermal relaxation, the temperature evolutions in the graphene/2D-SiC heterobilayer with a size of 100 × 10 nm^2^ are illustrated in Fig. 11. When the system is in thermal equilibrium, an ultrafast thermal impulse of 18 × 10^12^ W/m^2^ was imposed on the graphene layer for 50 fs at *T* = 300 K. The temperature of graphene ${T}_{GRA}$ rises to ~ 680 K while the temperature of 2D-SiC ${T}_{2D-SiC}$ remains the same at 300 K during the heat impulse process. After removal of the thermal impulse, as time progresses, the graphene temperature decreases, while the 2D-SiC temperature increases, implying the transfer of heat from graphene to 2D-SiC. Since graphene exhibits higher heat capacity per area than that of 2D-SiC, the ultimate equilibrium temperature of the graphene/2D-SiC heterobilayer is similar to the initial temperature of graphene. The ultimate temperature at the equilibrium state in the heterobilayer is ~ 425 K. The evolution of energy in the graphene layer is presented in Fig. 11. A least squares fitting of the energy evolution of graphene through Eq. (6) is also shown by the orange line in Fig. 11. The fitted curve is well able to describe the MD simulation, which demonstrates the validity of the TPP process. The thermal interfacial resistance at 300 K is determined to be 2.71 × 10^−7^ km^2^/W, similar to other vdW heterobilayers^16,32,41,43,45,46,55^. An outline of the thermal resistance of typical 2D material interfaces is given in Table 1. It can be inferred from these results that *R* between graphene and 2D-SiC is greater than graphene/silicene, graphene/h-BN and graphene/MoS_2_ heterobilayer.

**Table 1:** A comparison of the thermal interface resistance for different 2D heterobilayers determined from MD simulation.

Materials	R ($\times {10}^{-7} {\mathrm{km}}^{2}/\mathrm{W })$	Method	References
Graphene/stanine	~ 1.7–3.5	TPP	Hong et al.^41^
Graphene/${\mathrm{C}}_{3}\mathrm{N}$	~ 0.4–1.5	TPP	Han et al.^80^
Graphene/phosphorene	~ 0.85–1.75	TPP	Hong et al.^32^
Graphene/${\mathrm{MoS}}_{2}$	~ 0.9–1.8	TPP	Liu et al.^45^
Graphene/Si	~ 0.9–3.0	TPP	Yousefzadi et al.^81^
Graphene/*h*-BN	~ 1.5–4.96	LCM	Li et al.^48^
Graphene/silicene	~ 0.45–1.7	TPP	Liu et al.^16^
Graphene/2D-SiC	~ 1.59–2.71	TPP	Present study

*LCM* lumped capacity method.

**Figure 12:**
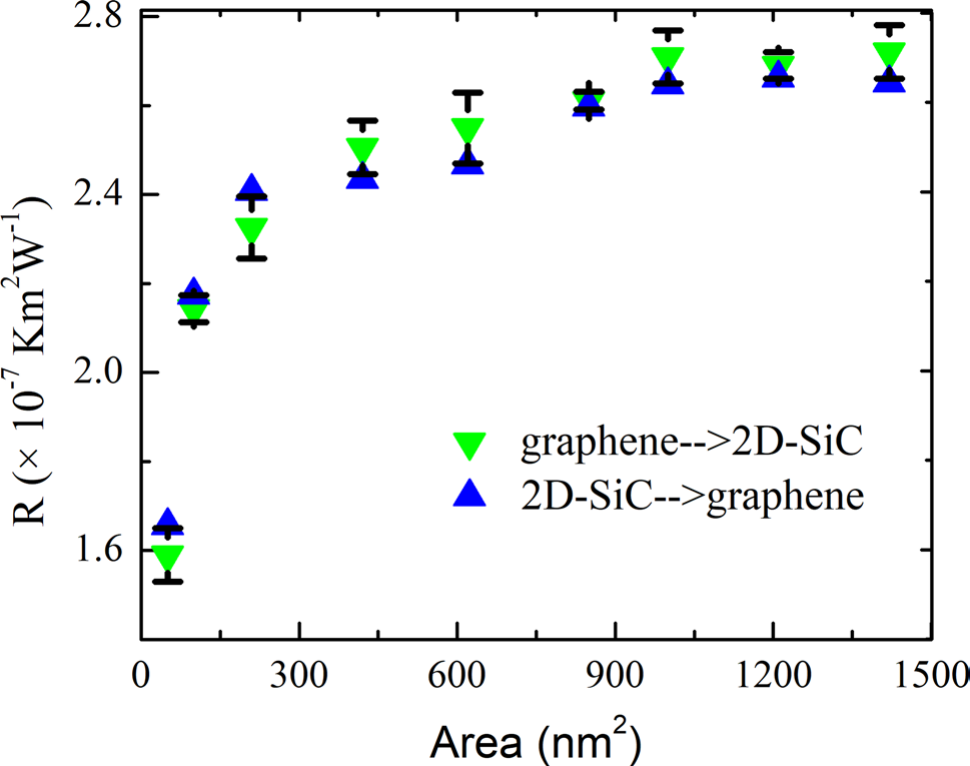
Interfacial thermal resistance of the graphene/2D-SiC heterobilayer for both graphene to 2D-SiC and 2D-SiC to graphene projection with variation of heterobilayer area from 50 to 1420 nm^2^. Fitted data of graphene to 2D-SiC corresponds well with the MD simulation results.

The thermal conduction at the interface between two different solids strongly depends on the direction of the heat current especially for dissimilar materials, which is known as a thermal rectification characteristic. We also calculated the *R* along the graphene → 2D-SiC and 2D-SiC → graphene directions to expose the thermal modification behavior in the graphene/2D-SiC heterostructure. Moreover, we take different lengths of 5.10, 10.2, 22.42, 42.10, 62.31, 85.55, 100, 121.1 and 142.2 nm with a fixed width of 10.8 nm to explore the impact of system area on *R*. Figure 12 shows the area dependence of *R* at *T* = 300 K. Firstly, it is found that the measured *R* is almost the same in both the graphene → 2D-SiC and 2D-SiC → graphene directions for a fixed heterobilayer area, which demonstrates the absence of the thermal rectification effect in the heterostructure. As a reference, with a system size of $100\times 10.8$ nm^2^, ${R}_{GRA\to 2D-SiC}$ and ${R}_{2D-SiC\to GRA}$ are found to be 2.71 × 10^−7^ km^2^/W and 2.65 × 10^−7^ km^2^/W, respectively, revealing only 2.21% difference. Moreover, the value of *R* was observed to upsurge monotonically with growing system size. As the system area enlarges, *R* rises initially, and then the rate of increase slows down from 454 nm^2^. And finally, *R* remains almost constant at $\sim 2.72\times {10}^{-7}$ km^2^/W from area 1080 nm^2^. The maximum *R* increases to 70.4% for increasing system area from 50 to 1420 nm^2^.

**Figure 13:**
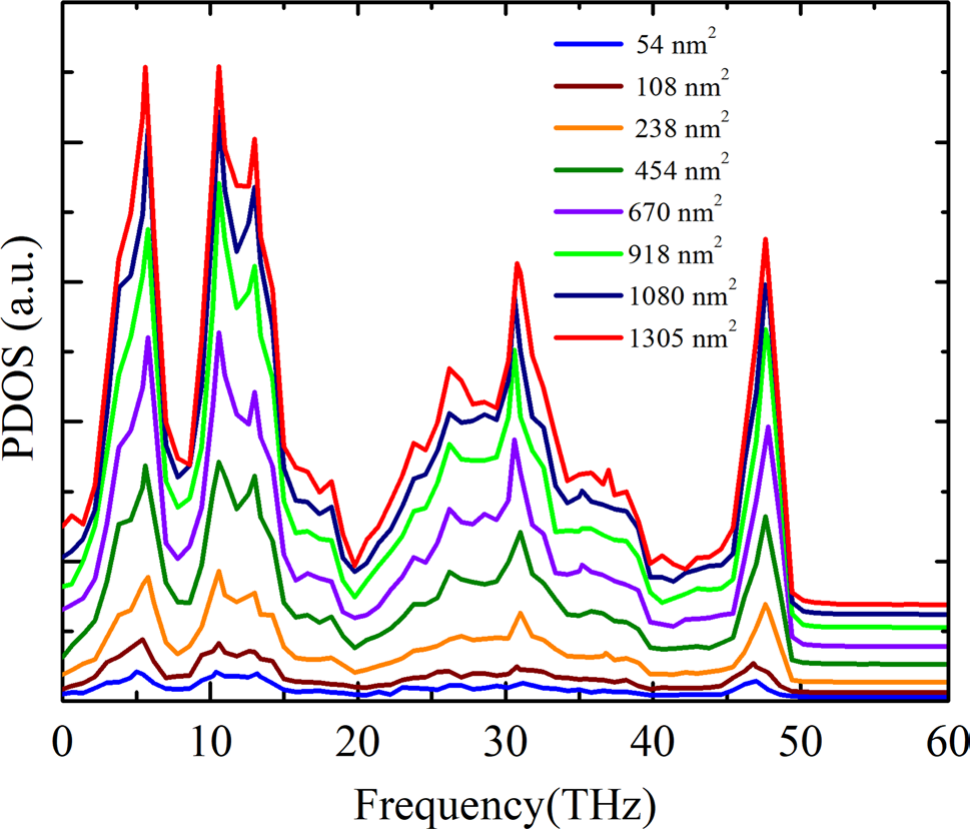
Out-of-plane PDOS of the graphene/2D-SiC heterobilayer for varying lengths from 5 to 121 nm with a fixed-width of 10.8 nm at 300 K.

**Figure 14:**
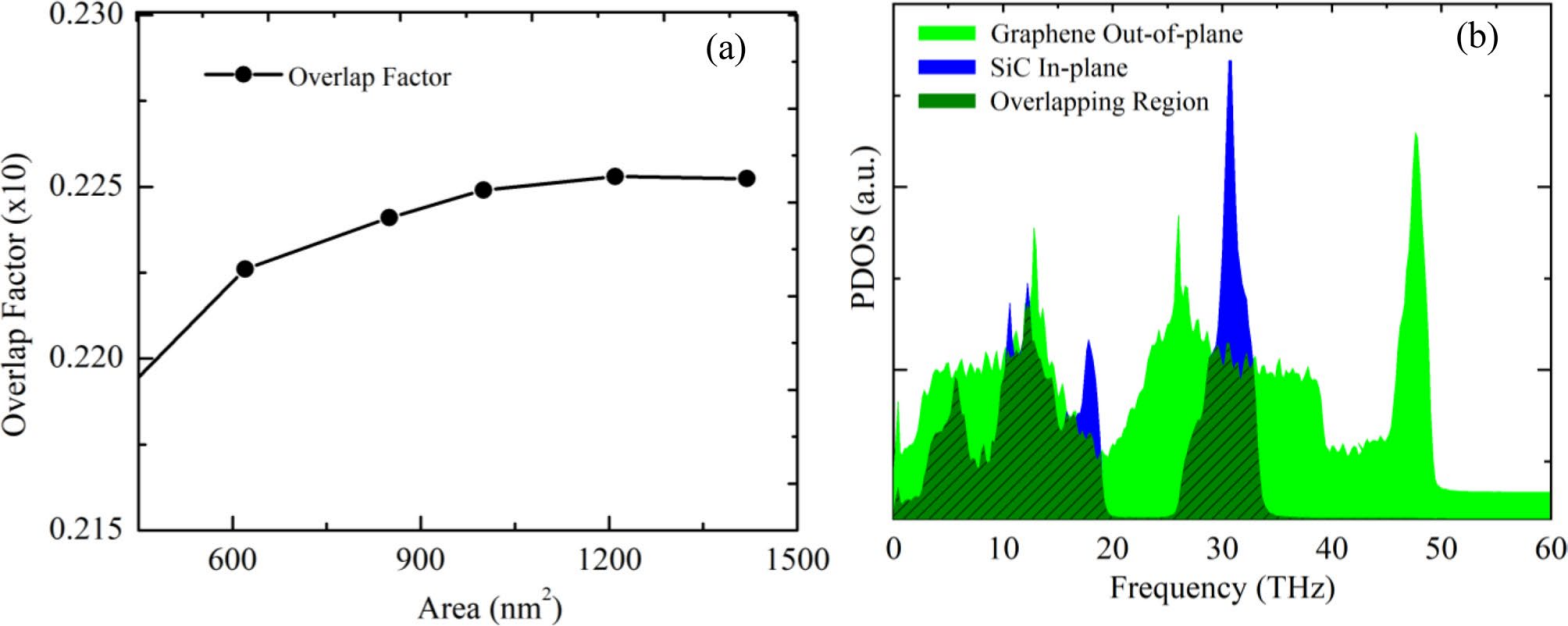
(**a**) Calculated overlap factor *S* as a function of the heterobilayer area. (**b**) PDOS of graphene Out-of-plane and SiC In-plane of $100\times 10.8$ nm^2^ graphene/2D-SiC vdWHs. The slant areas (olive) denote the overlap region between graphene out-of-plane and SiC in-plane.

To clarify the variation of *R* with system size, we calculated the ZA phonon of the heterobilayer for various system sizes because the ZA phonon dominantly contributes to the out-of-plane thermal conduction in graphene related materials. Figure 13 exhibits the out-of-plane PDOS of a graphene/2D-SiC vdWH as a function of the area of the heterobilayer. As the system area increases, improved behavior is perceived in the PDOS peaks owing to the enhancement of the acoustic mode phonons. As the ZA phonon mode transports from one layer to another supported layer, it scatters with the supported layer acoustic mode phonons (LA, TA, and ZA phonons) and develops thermal resistance between the interfaces. Therefore, as the amount of the ZA phonon mode rises, phonon scattering between the interfaces rises which causes the enhancement of the interface thermal resistance. Furthermore, Fig. 12 reveals that the *R* increases rapidly up to area 454 nm^2^, slows down from area 454 nm^2^ and then stays almost constant from area 1080 nm^2^. This indicates that the *R* is strongly size-dependent. In order to better illustrate and analyze the size-dependent phenomena of *R*, we also quantify the overlap factor *S* for various heterobilayer areas. The *S* mainly quantifies the amount of phonon overlapping between the interlayer as well as indicating the interlayer energy exchange efficiency. We quantify the *S* between the graphene out-of-plane (ZA) phonon modes and 2D-SiC in-plane (LA and TA) phonon modes as we apply an ultrafast heat impulse from graphene to 2D-SiC. The *S* between out-of-plane and in-plane PDOS is calculated using the following relation as^79^

12 $$S=\frac{{\int }_{0}^{\infty }{F}_{out}\left(\omega \right)\cdot {F}_{in}\left(\omega \right)d\omega }{{\int }_{0}^{\infty }{F}_{out}\left(\omega \right)d\omega \cdot {\int }_{0}^{\infty }{F}_{in}\left(\omega \right)d\omega }$$

where subscripts “out” and “in” represent graphene out-of-plane (ZA) and 2D-SiC in-plane (LA and TA) phonon modes. The overlap factor for varying heterobilayer areas is represented in Fig. 14a, and the overlapping region is represented in Fig. 14b. As the area rises, the *S* rises gradually to 850 nm^2^, and from 1080 nm^2^ it is almost constant. This implies the overlapping of phonons, as well as the energy exchange efficiency between the interlayer, increases up to 850 nm^2^ and appears to slightly decrease the scattering of phonons between the interlayer. As a result, the overall thermal resistance of the interface slows down from 454 to 850 nm^2^ areas. On the other hand, from 1080 nm^2^ the overlapping of phonons as well as the energy exchange capacity is almost constant, which means that from 1080 nm^2^ phonon scattering between the interlayer is almost constant. Consequently, the *R* remains almost constant from 1080 nm^2^ because it relies explicitly on the phonon scattering between the interlayer.

## Conclusions

The lateral and cross-plane thermal conductivity of the graphene/2D-SiC heterobilayer were systematically studied using the RNEMD simulation and TPP method, respectively. The original and optimized Tersoff potentials were used to illustrate their effect on the graphene/2D-SiC heterobilayer thermal conductivity. The optimized Tersoff potential provides better thermal conductivity estimation than the original Tersoff potential due to the correctly parameterized interatomic forces. A high thermal conductivity of ~ 452 W/m-K was obtained for the 100 nm length and 10.8 nm width heterobilayer at room temperature using the optimized Tersoff potential, superior to most other graphene based vdWHs such as graphene/MoS_2_, graphene/MoSe_2_, graphene/stanene, silicene/graphene, graphene/C_3_N, as well as individual 2D-SiC, phosphorene, hexagonal boron nitride (*h*-BN), MoS_2_ and MoSe_2_ of the same system size. The in-plane thermal conductivity of graphene, 2D-SiC and heterobilayer at infinite length were determined as 2239.49 W/m-K, 430.19 W/m-K and 1451.59 W/m-K, respectively. The total PDOS revealed that the active phonon frequencies in 2D-SiC are only 0–35 THz with a low phonon density in the high-frequency region, strictly bounding the lateral thermal conductivity to a lower value than graphene. With a system size of $100\times 10$ nm^2^, we estimated that out-plane thermal resistance of graphene → 2D-SiC as 2.71 × 10^−7^ km^2^/W and 2D-SiC → graphene as 2.65 × 10^−7^ km^2^/W at 300 K, respectively, indicating the absence of thermal rectification in the heterobilayer. Out-of-plane thermal resistance was found to expand monotonically with growing system size. The maximum R increases to 70.4% for increase of the system area from 50 to 1420 nm^2^. We found that the increase of the ZA phonon peak with the length caused the monotonic increase of the out-of-plane thermal resistance with system size. The superior thermal conduction, along with good electrical properties, could make the graphene/2D-SiC heterobilayer appealing as a high-performance thermal interface material or electronic material for next generation nanoelectronics.

## Data Availability

The data that support the findings of this study are available from the corresponding author upon reasonable request.
